# Tolerance to lens tilt and decentration of two multifocal intraocular lenses: using the quick contrast sensitivity function method

**DOI:** 10.1186/s40662-022-00317-y

**Published:** 2022-12-01

**Authors:** Dongling Guo, Jiaqi Meng, Keke Zhang, Wenwen He, Shiyu Ma, Zhong-lin Lu, Yi Lu, Xiangjia Zhu

**Affiliations:** 1 Eye Institute and Department of Ophthalmology, Eye & ENT Hospital, Fudan University, Shanghai, 200031 China; 2 NHC Key Laboratory of Myopia, Fudan University, Shanghai, China; 3 Key Laboratory of Myopia, Chinese Academy of Medical Sciences, Shanghai, China; 4 Shanghai Key Laboratory of Visual Impairment and Restoration, Shanghai, China; 5 State Key Laboratory of Medical Neurobiology, Fudan University, Shanghai, China; 6 Division of Arts and Sciences, NYU Shanghai, Shanghai, China; 7 Center for Neural Science and Department of Psychology, New York University, New York, USA; 8 NYU-ECNU Institute of Brain and Cognitive Science at NYU Shanghai, Shanghai, China

**Keywords:** qCSF, Contrast sensitivity, IOL, Tilt, Decentration

## Abstract

**Background:**

Quick contrast sensitivity function (qCSF) method is an advanced quick method for contrast sensitivity function (CSF) evaluation. This study evaluated the contrast sensitivity (CS) of eyes undergoing cataract surgery with multifocal intraocular lens (IOL) implantation and its tolerance to IOL tilt and IOL decentration using the qCSF method.

**Methods:**

Patients undergoing uneventful phacoemulsification and a trifocal IOL (Zeiss AT LISA tri 839MP, Carl Zeiss, Germany) or an extended depth-of-focus (EDOF) IOL (Tecnis Symfony ZXR00, Johnson & Johnsons, USA) implantation were included. Monocular contrast sensitivity was measured using the qCSF method at one month post-surgery. IOL tilt and decentration were measured using an optical aberrometer (OPD-Scan III, NIDEK, Japan).

**Results:**

Seventy-two patients/eyes with the 839MP IOL and 64 patients/eyes with the ZXR00 IOL were included. Area under the log CSF (AULCSF) and CS acuity did not differ significantly between the two groups. The ZXR00 IOL group showed better CS at 1 cpd (1.137 ± 0.164 vs. 1.030 ± 0.183 logCS) and 1.5 cpd (1.163 ± 0.163 vs. 1.071 ± 0.161 logCS), while the 839MP IOL group had better CS at 6 cpd (0.855 ± 0.187 vs. 0.735 ± 0.363 logCS). In the 839MP IOL group, all CSF metrics were negatively correlated with IOL tilt (all *P* < 0.05), while in the ZXR00 IOL group, the CS at 3 cpd had no significant correlation with IOL tilt (*P* > 0.05). Among myopic eyes, fewer CSF metrics were negatively correlated with IOL tilt in the ZXR00 IOL group than in the 839MP IOL group. No significant correlation was found between CSF metrics and IOL decentration.

**Conclusions:**

The ZXR00 and the 839MP IOL groups presented comparable CSF. CS was negatively correlated with IOL tilt, instead of decentration in multifocal IOLs, particularly among myopic eyes. The ZXR00 IOL had better tolerance to IOL tilt in myopic eyes.

## Background

Multifocal intraocular lenses (IOLs), which can provide clear visual acuity at different distances, have been enjoying growing popularity among cataract patients [1]. However, loss of contrast sensitivity (CS) is one of the major concerns regarding multifocal IOLs [2, 3]. Previous studies also revealed that some metrics such as aberrations and modular transfer function (MTF) can be affected by IOL tilt and decentration after multifocal IOL implantation [4–7]. To our knowledge, no study has investigated the influence of IOL tilt and decentration on CS among patients implanted with multifocal IOLs. Therefore, assessing the correlation between CS and IOL tilt or IOL decentration may be helpful to determine the CS loss attributed to IOL tilt and decentration in multifocal IOLs.

The traditional tools for measuring CS, such as the Pelli-Robson chart, the Vistech chart, and the Functional Acuity Contrast Test (FACT) have shortcomings. The Pelli-Robson chart is based on a recognition visual acuity test, but its low spatial frequency and three-letter contrast increments could contribute to a learning effect after a few usages [8, 9]. Five spatial frequencies and nine contrast levels make up the FACT [10]. Some researchers have offered different strategies to segregate the parvocellular (PC) and magnocellular (MC) pathways for psychophysical CS assessments. They were supposed to identify the psychophysical signature of the PC and MC pathways, but they took too long (around 12 h) for therapeutic use [11, 12]. These limitations hinder their clinical application, especially in cases requiring distinction of subtle differences. Other new tools have also been proposed for testing multifocal IOLs, either measuring CSF at a single distance from far to near (ClinicCSF) or the CS at multiple distances (Multifocal Lens Analyzer) [13, 14]. Despite the advantages versus conventional tests, they also have a limited number of spatial frequencies and contrast levels. Incidentally, the quick contrast sensitivity function (qCSF) method is a novel method for contrast sensitivity function (CSF) evaluation embedded in the Manifold Contrast Vision Meter (Adaptive Sensory Technology, San Diego, CA, USA). It can assess the entire CSF rapidly in 25 trials (about 5–10 min), which even though requires longer testing times than the previous mentioned CSF clinical tests (about 2–3 min) [13], avoids the limitation of using discrete steps for contrast and frequencies by means of utilizing a Bayesian active learning procedure [15].

Here, we compared the CSF of patients implanted with a trifocal IOL and an extended depth-of-focus (EDOF) IOL using the qCSF method, and investigated their tolerance to IOL tilt and decentration. The effect of age on CSF was also evaluated.

## Methods

This retrospective study was approved by the institutional review board of the Eye & Ear, Nose, and Throat (ENT) Hospital of Fudan University, Shanghai, China, and was registered at www.clicicaltrials.gov (accession number NCT02182921). All procedures adhered to the tenets of the Declaration of Helsinki. All participants provided written informed consent before cataract surgery for the use of their clinical data.

### Subjects

Patients underwent uneventful cataract surgery with a trifocal IOL (Zeiss AT LISA tri 839MP, Carl Zeiss, Germany) or an EDOF IOL (Tecnis Symfony ZXR00, Johnson & Johnsons, USA) implantation and were continuously recruited from the Eye & ENT Hospital of Fudan University between September 1, 2021, and February 1, 2022. Eyes with corneal pathologies including glaucoma, uveitis, zonular weakness, strabismus, retinal pathologies, had intraoperative or postoperative complications, previous trauma or surgeries, and incomplete clinical data were excluded. One eye was randomly selected if both eyes met the criteria. Finally, a total of 136 eyes from 136 patients were included, with 72 eyes implanted with the 839MP IOL and 64 eyes implanted with the ZXR00 IOL. Eyes with axial length > 24.5 mm were defined as myopic eyes, and eyes with axial length ≤ 24.5 mm were defined as non-myopic eyes.

### Preoperative examinations

All patients received complete ophthalmic examinations before the operation, including visual acuity assessment, slit lamp examination, fundoscopy, B-scan ultrasonography, corneal topography (Pentacam HR, Oculus Optikgeräte, Wetzlar, Germany), and axial length (AL) measurements (IOLMaster 700, Carl Zeiss AG, Oberkochen, Germany).

### Surgical procedure

All the surgeries were performed by a single, experienced surgeon (XZ) using the standard procedure. A 2.6 mm clear corneal incision was made temporally before a 5.5 mm continuous curvilinear capsulorhexis, hydrodissection, and phacoemulsification. The IOL was implanted in the capsular bag and adjusted to the center. A capsular tension ring was inserted in eyes with AL > 28.0 mm to keep the actual effective lens position. After thorough removal of the viscoelastic, the incision was hydrated. All patients were prescribed with routine postoperative medications, including 0.5% levofloxacin (Cravit, Santen, Japan), 1% prednisolone acetate (Pred Forte, Allergan, Ireland), and pranoprofen (Pranopulin, Santen, Japan) eye drops.

### Postoperative examinations

One month after surgery, all patients underwent ophthalmic examinations, including visual acuity assessment, CSF measurement, and IOL tilt and decentration evaluation.

Uncorrected distance, intermediate and near visual acuity [UDVA, UIVA, UNVA, logarithm of the minimal angle of resolution (logMAR)] and corrected distance visual acuity (CDVA, logMAR) were recorded monocularly at 4 m, 60 cm and 40 cm, respectively, using an Early Treatment Diabetic Retinopathy Study (ETDRS) chart (Wehen Vision Technology Co., Ltd, Guangzhou, China) at high contrast (96%) under photopic conditions (85 cd/m^2^).

CSF was measured using the qCSF procedure on the Manifold Contrast Vision Meter (Adaptive Sensory Technology, San Diego, CA, USA), a novel computerized method for assessing contrast sensitivity. The platform consisted of a 46″ light emitting diode (LED) screen of 1920 × 1080 pixels, calibrated to 90 cd/m^2^ background luminance. At a viewing distance of 3 m, under mesopic conditions (3 cd/m^2^), the screen displays three bandpass-filtered Sloan numeric optotypes at a time. Patients wearing best-correcting spectacles were asked to patch the non-test eye. Patients’ responses (correct, incorrect, or number not seen) were recorded by a trained operator using a mobile phone for each number [16]. During 25 triplets, the qCSF distributes testing across a wide range of contrast (0.002% to 100%) and spatial frequencies [from 1 to 27 cycle per degree (cpd)] using a Bayesian optimal strategy to best predict the CSF shape [17]. Test results, including the area under the log CSF (AULCSF), CSF acuity (spatial frequency where threshold contrast is 100%), and CS at 1, 1.5, 3, 6, 12, 18 cpd, were recorded and used for analysis.

IOL tilt and decentration were evaluated using an optical aberrometer (OPD-Scan III, NIDEK, Japan). The tilt of the IOL was obtained directly from the aberrometer in the wavefront mode under a 4.0 mm pupil diameter, following pupil dilation with a mixture of 0.5% phenylephrine and 0.5% tropicamide (1% Mydrin-P, Santen, Japan). The overall decentration was the distance between the center of the multifocal IOL (also the center of diffractive rings) and the visual axis in the retrobulbar illumination analysis mode, which was then decomposed into horizontal and vertical decentrations. Positive values indicated the nasal and superior decentration, while negative values indicated the opposite decentration. More detailed description about the measurement of IOL tilt and decentration can be found in our previous report [18].

### Statistical analysis

Quantitative data were expressed as mean ± standard deviations (SD) and compared using the Student’s t-test. Categorical data were displayed as proportions and compared using the χ^2^ test. Correlations between CSF parameters and IOL tilt, IOL decentration or age were assessed using the Pearson’s correlation analysis. Backward stepwise multiple linear regression analyses were performed for predictors of CS at 1, 6 and 18 cpd. A *P* value < 0.05 was considered statistically significant. All analyses were performed using SPSS (version 20.0, IBM Corp., New York, US).

## Results

### Characteristics

The demographic characteristics of the included patients are shown in Table 1. There were no statistically significant differences between the two groups except postoperative UNVA, which was better in the 839MP group (all *P* > 0.05 except post-UNVA, *P* = 0.01).

**Table 1:** Demographics of participants

Characteristics	839MP (n = 72)	ZXR00 (n = 64)	*P* value
Age (years)	61.21 ± 9.69	63.58 ± 10.15	0.17
Sex (male/female)	35/37	32/32	0.87
Eye laterality (right/left)	39/33	30/34	0.39
AL (mm)	25.20 ± 2.04	24.88 ± 1.57	0.30
IOL power (D)	16.01 ± 5.60	17.07 ± 4.6	0.21
Pre-CDVA (logMAR)	0.48 ± 0.26	0.51 ± 0.28	0.52
Post-UDVA (logMAR)	0.09 ± 0.10	0.08 ± 0.12	0.68
Post-CDVA (logMAR)	0.04 ± 0.05	0.04 ± 0.08	0.81
Post-UIVA (logMAR)	0.12 ± 0.04	0.09 ± 0.02	0.19
Post-UNVA (logMAR)	0.07 ± 0.02	0.15 ± 0.03	0.01*

*IOL* = intraocular lens; *D* = diopter; *logMAR* = logarithm of the minimal angle of resolution; *pre-CDVA* = preoperative corrected distance visual acuity; *post-UDVA* = postoperative uncorrected distance visual acuity; *post-CDVA* = postoperative corrected distance visual acuity; *post-UIVA* = postoperative uncorrected intermediate visual acuity; *post-UNVA* = postoperative uncorrected near visual acuity

*Statistically significant (*P* < 0.05) values

### Postoperative contrast sensitivity function

**Fig. 1:**
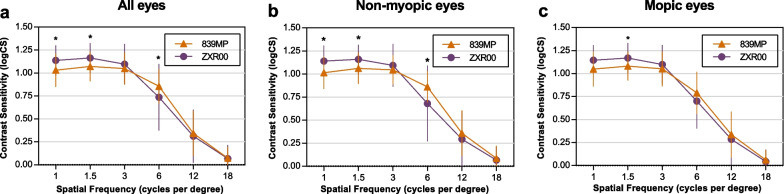
Monocular distance contrast sensitivity functions of the 839MP IOL group and the ZXR00 IOL group. Comparisons of monocular distance contrast sensitivity function of all eyes (**a**), non-myopic eyes (**b**), and myopic eyes (**c**) in the 839MP IOL group and the ZXR00 IOL group. Data are expressed as mean ± 95% confidence interval. *P < 0.05

No significant differences in AULCSF and CSF acuity were found between the 839MP and the ZXR00 IOL groups (AULCSF: 0.83 ± 0.19 vs. 0.83 ± 0.24, respectively; CSF acuity: 16.38 ± 5.10 vs. 15.06 ± 6.25, respectively; both *P* > 0.05). Among all the included eyes, CS was significantly higher at 1 and 1.5 cpd in the ZXR00 IOL group, and significantly higher at 6 cpd in the 839MP IOL group (Fig. 1a, all *P* < 0.05). The results of the non-myopic eyes were similar to the overall results (Fig. 1b**,** all *P* < 0.05). However, among myopic eyes, significant CS difference was only found at 1.5 cpd between the 839MP and the ZXR00 IOL groups (Fig. 1c, *P* = 0.027). Furthermore, Pearson’s correlation analysis indicated an age-related decline in contrast sensitivity, with AULCSF, CSF acuity and CS at 6, 12, 18 cpd negatively related to age (r =  − 0.217, − 0.227, − 0.249, − 0.170, − 0.194, respectively, all *P* < 0.05).

### Postoperative IOL tilt and decentration

At one month after surgery, the IOL tilt in this study ranged from 0.02 to 0.71 µm, and the total IOL decentration ranged from 0.01 to 0.79 mm. No significant difference of IOL tilt and total IOL decentration was found between the 839MP and the ZXR00 IOL groups (tilt: 0.23 ± 0.14 and 0.24 ± 0.14 µm, respectively, *P* = 0.582; decentration: 0.25 ± 0.18 and 0.26 ± 0.14 mm, respectively, *P* = 0.271). Likewise, no significant differences in IOL tilt and total IOL decentration were found between myopic and non-myopic eyes (tilt: 0.23 ± 0.14 and 0.23 ± 0.13 µm, respectively, *P* = 0.788; decentration: 0.22 ± 0.17 and 0.19 ± 0.15 mm, respectively, *P* = 0.455). Detailed comparisons of the non-myopic and myopic eyes are shown in Table 2. In non-myopic eyes, postoperative IOL tilt and decentration did not differ significantly between the two groups (all *P* > 0.05). In myopic eyes, the ZXR00 IOL group had significantly greater inferior vertical decentration than the 839MP IOL group (*P* < 0.05).

**Table 2:** Postoperative intraocular lens (IOL) tilt and decentration

Tilt or decentration	Non-myopic eyes			Myopic eyes		
	839MP (n = 36)	ZXR00 (n = 32)	*P* value	839MP (n = 36)	ZXR00 (n = 32)	*P* value
Tilt (μm)	0.23 ± 0.15	0.24 ± 0.13	0.70	0.22 ± 0.13	0.23 ± 0.14	0.70
Overall decentration (mm)	0.23 ± 0.18	0.21 ± 0.15	0.40	0.16 ± 0.16	0.27 ± 0.15	0.07
Vertical decentration (mm)	0.04 ± 0.23	0.04 ± 0.19	0.91	0.01 ± 0.22	− 0.14 ± 0.18	0.004*
Horizontal decentration (mm)	0.03 ± 0.25	0.00 ± 0.23	0.63	− 0.06 ± 0.24	0.02 ± 0.25	0.20

Data expressed as mean ± standard deviation

*Statistically significant (*P* < 0.05) values

### Influence of IOL tilt and decentration on CSF

The influence of IOL tilt on CSF is displayed in Table 3. In the 839MP IOL group, AULCSF, CSF acuity and CS at all spatial frequencies were negatively correlated with IOL tilt (all *P* < 0.05). In the ZXR00 IOL group, only AULCSF, CSF acuity and CS at 1 and 1.5 cpd were negatively correlated with IOL tilt (all *P* < 0.05). Among myopic eyes, significant negative correlations were found between AULCSF, CSF acuity, CS at 1, 1.5, 3, 12, 18 cpd and IOL tilt in the 839MP IOL group, while in the ZXR00 IOL group, only CS at 1.5 cpd was negatively correlated with IOL tilt. No significant correlation was found among non-myopic eyes in both IOL groups. None of the CSF metrics was significantly correlated to overall or horizontal IOL decentration in both IOL groups (all *P* > 0.05).

**Table 3:** Correlation coefficients between intraocular lens (IOL) tilt and contrast sensitivity function (CSF) metrics

Parameters	All eyes				Non-myopic eyes				Myopic eyes			
	839MP (n = 72)	*P* value	ZXR00 (n = 64)	*P* value	839MP (n = 36)	*P* value	ZXR00 (n = 32)	*P* value	839MP (n = 36)	*P* value	ZXR00 (n = 32)	*P* value
AULCSF	− 0.366	0.002*	− 0.253	0.046*	− 0.315	0.06	− 0.276	0.13	− 0.43	0.009*	− 0.242	0.19
CSF acuity	− 0.314	0.007*	− 0.247	0.049*	− 0.158	0.36	− 0.245	0.18	− 0.452	0.006*	− 0.251	0.17
1 cpd	− 0.216	0.03*	− 0.296	0.02*	− 0.086	0.62	− 0.346	0.05	− 0.42	0.01*	− 0.262	0.15
1.5 cpd	− 0.315	0.007*	− 0.327	0.008*	− 0.138	0.42	− 0.249	0.17	− 0.46	0.005*	− 0.431	0.01*
3 cpd	− 0.346	0.003*	− 0.188	0.14	− 0.284	0.09	− 0.220	0.23	− 0.418	0.01*	− 0.173	0.36
6 cpd	− 0.338	0.004*	− 0.242	0.054	− 0.242	0.16	− 0.276	0.13	− 0.3	0.08	− 0.327	0.07
12 cpd	− 0.290	0.01*	− 0.175	0.17	− 0.115	0.50	− 0.178	0.33	− 0.417	0.01*	− 0.148	0.42
18 cpd	− 0.349	0.003*	− 0.130	0.31	− 0.302	0.07	− 0.200	0.27	− 0.387	0.02*	− 0.016	0.93

*CSF* = contrast sensitivity function; *AULCSF* = area under the log CSF; *cpd* = cycles per degree

*Statistically significant (*P* < 0.05) values

### Multivariate analysis: variables associated with contrast sensitivity

Backward stepwise multiple linear regression analyses were performed for CS at low (1 cpd), median (6 cpd) and high (18 cpd) spatial frequencies, using sex, eye laterality, IOL type, age, AL, IOL tilt and IOL decentration as predictors. Significant predictors for worse CS at 1 cpd were the 839MP IOL group and interaction between IOL tilt and IOL decentration (standardized coefficient β = 0.297 and − 0.325, respectively, both *P* < 0.001, r^2^ = 0.186). Simple effect analysis showed that increasing decentration level led to a greater negative effect of IOL tilt on CS at 1 cpd. The CS at 6 and 18 cpd were negatively correlated with age and IOL tilt (6 cpd: β = − 0.176 and − 0.209, respectively, both *P* < 0.05, r^2^ = 0.076; 18 cpd: β = − 0.141 and − 0.248, respectively; both *P* < 0.05, r^2^ = 0.082).

## Discussion

With recent developments in surgical techniques and IOLs, we have entered a new era of refractive cataract surgery, providing better visual quality and spectacle independence for patients. Trifocal and EDOF IOLs are now widely used [19, 20]. However, IOL tilt and decentration can lower patients’ satisfaction by deteriorating various intraocular objective and perceptual parameters [21, 22]. To our knowledge, no study has investigated the influence of IOL tilt and decentration on CS among patients implanted with multifocal IOLs. In this study, we compared the CSF between cataract patients implanted with a trifocal IOL and an EDOF IOL using the newly developed qCSF method, and assessed the influence of IOL tilt and decentration on CSF. We found that (1) the two groups obtained comparable overall CSF, (2) CSF was negatively correlated with IOL tilt, but not with IOL decentration, especially in myopic eyes, and (3) the ZXR00 IOL group exhibited better tolerance to IOL tilt in myopic eyes.

The qCSF on the Manifold Contrast Vision Meter platform was for the first time applied to the follow-up evaluation of cataract patients implanted with multifocal IOLs in this study. Compared to conventional CSF tests, the newly developed qCSF method dramatically improved the measurement range and accuracy by applying a Bayesian information maximization rule [23]. It could evaluate CS across all the spatial frequencies rapidly (about 5–10 min) with excellent precision and accuracy [15], whereas the traditional Pelli-Robson chart can only assess CS at one spatial frequency [9]. The qCSF has been proven to be a sensitive way to detect early-stage diabetic retinopathy, an ideal model to classify amblyopia, and a useful metric to assess glaucomatous visual function deficiency [17, 24, 25]. With this tool, we found no significant difference in overall CSF metrics (AULCSF and CSF acuity) between the 839MP IOL group and the ZXR00 IOL group. Our results provide a further verification and a valuable complement to previous studies applying traditional CSF methods.

We found significant CS differences between the two IOL groups at low and medium spatial frequencies. The ZXR00 IOL group exhibited higher CS at 1 and 1.5 cpd, which was consistent with Mencucci et al.’s study [26]. The higher CS at low spatial frequencies of the ZXR00 IOL might because of its elongated focal point created by the unique echelette design, the added compensations for chromatic and spherical aberration of the cornea by the achromatic diffractive design, and the aspheric design [27]. On the other hand, the 839MP IOL group had better CS at 6 cpd. The 839MP IOL has a diffractive structure and an anti-posterior capsule opacification (PCO) barrier shape that could reduce the effect of PCO on CS [28]. In addition, the 839MP IOL uses a smooth topographical structure (SMP) technology for its surface without any right angles, which could reduce light scattering and probably the reason of better CS at 6 cpd. However, the CSF differences were reduced in myopic eyes possibly due to myopic retinal function disruption impairing CS, especially in high spatial frequencies [29, 30].

Similar to previous studies, myopic eyes still exhibited slightly greater overall IOL decentration compared to emmetropic eyes [31, 32], and had significantly greater inferior IOL decentration in the ZXR00 IOL group than the 839MP IOL group [18]. As previously reported, the former can be explained by the bigger capsular bag size of myopic eyes, so that IOLs are more likely to “sink” which leads to greater inferior IOL decentration. The latter is probably because of the plate-haptic design of the 839MP IOL. Compared with the c-loop design IOL, the plate-haptic design omits the gap between the optic and haptics. Therefore, it gains greater support from the capsular bag through its four corners and has a better capsular stability in longer eyes. However, the correlation between CSF and IOL decentration was not significant, possibly because the relatively small decentration was not enough to result in clinically obvious CS deficiency [33].

It is noteworthy that CSF metrics were negatively correlated with IOL tilt in both groups. Most previous studies have focused on the impact of IOL tilt on objective indices such as HOAs and MTF [4–7]. Lawu et al. and Liu et al. found that IOL tilt increased wavefront aberrations and degraded optical performance [4, 34]. Montes-Mico et al. demonstrated that the MTFs for the refractive-diffractive IOL decreased when the IOL was tilted [6]. Therefore, we speculate that IOL tilt attenuates CSF by increasing wavefront aberrations and decreasing MTF values. Furthermore, the ZXR00 IOL group showed better tolerance to IOL tilt, which might also benefit from its EDOF design.

Interestingly, we found that IOL tilt had an interactive effect with IOL decentration at low CS frequency i.e., when IOL decentration increases, IOL tilt had a greater effect on CSF. We also confirmed that old age is another risk factor for worse CS at medium and high spatial frequencies, which was in accordance with previous studies [35, 36]. Finally, it was important to note that our CSF was underestimated for both lenses in comparison to previous published studies, owing to the use of spatial filtered numbers rather than sinusoidal gratings [37, 38]. A validation of this test is required for future studies with multifocal IOLs.

## Conclusions

In conclusion, the 839MP IOL and the ZXR00 IOL presented comparable AULCSF and CSF acuity measured by the qCSF method, while the ZXR00 IOL group presented better CS at low spatial frequencies, and the CS of the 839MP IOL group presented better CS at medium spatial frequencies. CSF was negatively correlated with IOL tilt, but not with decentration, particularly among myopic eyes. Moreover, the 839MP IOL showed lower vertical decentration and the ZXR00 IOL had better tolerance to IOL tilt in myopic eyes. Thus, both 839MP IOL and ZXR00 IOL may be suited for myopic eyes with higher risk of IOL decentration and tilt.

## Data Availability

The data that support the findings of this study are available from the corresponding author upon reasonable request.

## Abbreviations

IOL: Intraocular lens
CS: Contrast sensitivity
PC: Parvocellular
MC: Magnocellular
LED: Light emitting diode
CSF: Contrast sensitivity function
qCSF: Quick contrast sensitivity function
logMAR: Logarithm of the minimal angle of resolution
SD: Standard deviation
EDOF: Extended depth-of-focus
AULCSF: Area under the log CSF
MTF: Modular transfer function
FACT: Functional Acuity Contrast Test
AL: Axial length
UDVA: Uncorrected distance visual acuity
UIVA: Uncorrected intermediate visual acuity
UNVA: Uncorrected near visual acuity
CDVA: Corrected distance visual acuity
cpd: Cycles per degree
